# Seguimiento inmunológico después de trasplante renal: una propuesta para la práctica clínica en Colombia

**DOI:** 10.7705/biomedica.5092

**Published:** 2020-06-30

**Authors:** Melissa Andrea Ortiz, Cristiam Mauricio Álvarez, Ana María Arrunátegui, Yazmín Rocío Arias, Adriana Villegas

**Affiliations:** 1 Grupo VIREM, Departamento de Microbiología, Facultad de Salud, Universidad del Valle, Cali, Colombia Grupo VIREM Universidad del Valle Cali Colombia; 2 Unidad de Trasplantes, Hospital Universitario del Valle Evaristo García, Cali, Colombia Hospital Universitario del Valle Evaristo García Cali Colombia; 3 Laboratorio de Inmunología de Trasplantes, Grupo de Inmunología Celular e Inmunogenética, Instituto de Investigaciones Médicas, Facultad de Medicina, Universidad de Antioquia, Medellín, Colombia Universidad de Antioquia Grupo de Inmunología Celular e Inmunogenética Universidad de Antioquia Medellín Colombia; 4 Departamento de Patología y Medicina de Laboratorio, Fundación Valle del Lili, Cali, Colombia Fundación Valle del Lili Cali Colombia; 5 Centro Regulador de Trasplantes, Red Nacional de Donación y Trasplantes, Instituto Nacional de Salud, Bogotá, D.C., Colombia Centro Regulador de Trasplantes, Red Nacional de Donación y Trasplantes Instituto Nacional de Salud BogotáD.C Colombia; 6 Laboratorio de Inmunología de Trasplantes e Inmunogenética, Fundación Valle del Lili, Cali, Colombia Laboratorio de Inmunología de Trasplantes e Inmunogenética Fundación Valle del Lili Cali Colombia

**Keywords:** trasplante de riñón, isoanticuerpos, rechazo de injerto, Kidney transplantation, isoantibodies, graft rejection

## Abstract

El daño del injerto es un proceso multifactorial que se inicia tempranamente después de la mayoría de los trasplantes de donantes sin HLA idéntico. Puede deberse a las comorbilidades del receptor, al estado del donante, al tiempo de isquemia, y al fenómeno de isquemia y reperfusión, entre otros, condiciones que inducen factores metabólicos e inmunológicos que finalmente desembocan en la disfunción del injerto. Sin embargo, entre el momento del trasplante y la aparición de los signos y síntomas existe un periodo que puede tardar semanas o años. Por ello, después del trasplante renal, es importante hacer un seguimiento racional que incluya la evaluación clínica y permita anticiparse al daño inmunológico del injerto. En este ensayo se propone un algoritmo de seguimiento del injerto renal después del trasplante.

## Abstract (en)

Graft damage is a process that starts at the moment of transplantation, due to comorbidities of receptor, donor status, ischemia time, ischemia-reperfusion phenomenon, among others, those induce metabolic and immune factors that ultimately trigger clinical manifestations of graft dysfunction. However, the preclinical progression between the time of transplantation and the appearance of signs and symptoms of graft damage can take weeks to years. Therefore, the implementation of rational monitoring approaches during the posttransplantation period is critical and should include not only the clinical follow-up but also anticipate immunological graft damage. In the present essay, we propose an immunological monitoring algorithm for the post-renal transplantation period.

Si bien el uso de nuevos fármacos immunosupresores ha permitido disminuir la tasa de rechazo en los trasplantes, los efectos colaterales, como la alta incidencia de enfermedades infecciosas, el desarrollo de múltiples neoplasias y la afectación renal por la toxicidad asociada con los medicamentos, continúan siendo factores que disminuyen ostensiblemente la calidad de vida del paciente con trasplante, a la vez que incrementan los costos de su manejo después de realizado.

La detección temprana del proceso de rechazo y la eventual intervención que pueda requerirse constituyen algunos de los principales retos para los nefrólogos responsables del manejo del paciente con trasplante renal y su esfuerzo por evitar la pérdida del aloinjerto ^1^.

El daño del injerto es un proceso multifactorial que se inicia tempranamente en la mayoría de los trasplantes de donantes sin HLA. Puede deberse a las comorbilidades del receptor, el estado del donante, el tiempo de isquemia, y el fenómeno de isquemia y reperfusión, entre otros factores que inducen cambios metabólicos e inmunológicos, y dan lugar a manifestaciones clínicas de la disfunción del injerto ^2^. Sin embargo, entre el momento del trasplante y la aparición de los signos y síntomas, hay un periodo que puede tardar semanas o años, por lo cual es importante hacer un seguimiento racional después del trasplante renal que incluya la evaluación clínica y permita anticiparse al daño inmunológico del injerto ^1^^,^^3^^,^^4^. Tal y como lo han descrito Gosset, *et al.*, la presencia de anticuerpos anti-HLA específicos de donante (*donorspecific antibody*, DSA) es un importante aspecto determinante de la fibrosis prematura y acelerada del injerto, independientemente de los factores de riesgo tradicionales y de la reacción mediada por anticuerpos ^5^.

En este contexto, en el presente ensayo se busca comprender los eventos inmunopatológicos que llevan a la pérdida del injerto renal, con el fin de proponer una opción de seguimiento para detectar el daño tempranamente.

## El daño del injerto ocurre mucho tiempo antes del diagnóstico clínico y patológico del rechazo.

El rechazo renal de tipo celular implica cambios histopatológicos claros que permiten hacer un diagnóstico estratificado según lo han determinado los consensos de Banff llevados a cabo hasta el 2017. Por el contrario, no se cuenta con una metodología lo suficientemente sensible para el diagnóstico oportuno del rechazo renal de tipo humoral ^6^, sobre todo si se tiene en cuenta que el depósito de anticuerpos contra los antígenos del injerto aparece mucho antes de la lesión histológica franca y que la clásica detección del depósito de C4d (marcador de la activación del complemento por anticuerpos) puede estar presente o ausente al tratar de hacer el diagnóstico histopatológico del rechazo humoral ^3^^,^^7^^,^^8^.

Se sabe que la detección clínica del rechazo, e incluso la histopatológica, es tardía,y se acompaña de un probable daño irreversible del injerto que desemboca en su pérdida, en tanto que los hallazgos de la producción de anticuerpos por depósito de C4d en el tejido, o por la aparición de anticuerpos nuevos después del trasplante, DSA *de novo* (DSA*dn*), son más tempranos y eventualmente reversibles con el tratamiento adecuado y oportuno ^5^.

Desde comienzos de este siglo, se ha descrito la importancia de la aparición de anticuerpos anti-HLA específicos del donante en el daño renal a partir del implante y su impacto en la supervivencia del injerto y en la tasa de rechazo agudo ^6^.

En cuanto a la frecuencia, el rechazo agudo con anticuerpos DSA *de novo* (DSA*dn*) es del 1 al 6 % y aumenta del 21 al 55 % en pacientes con presencia de DSA anterior al trasplante ^5^. Hasta un 15 % de los pacientes con trasplante renal desarrolla DSA*dn* al año y, el 96 %, a los cinco años, con una media de aparición de 4,6 años. Además, los DSA de clase I se han asociado con el rechazo temprano, en tanto que los de clase II (*locus* DR y DQ) son los de peor pronóstico para el rechazo crónico (DR) y la disfunción del injerto; estos tienden a aparecer más temprano dependiendo del cumplimiento del tratamiento por parte del paciente.

Con respecto al impacto, muchos grupos de investigación en trasplantes han demostrado que la aparición, la persistencia o el incremento de DSA en los pacientes con trasplante se correlacionan con la poca supervivencia del injerto ^11^. Se ha demostrado que la persistencia o el incremento de los DSA en suero, el desarrollo de DSA*dn* y la presencia de DSA de clase II, se correlacionan con la poca supervivencia del injerto y una mayor tasa de rechazo agudo. En el lapso de los primeros cinco años a partir de la detección de los DSA, el 50 % de los pacientes en el estudio de Hidalgo perdió sus injertos ^12^. Wiebe, *et al.*, encontraron un aumento en la pérdida del injerto en pacientes que desarrollaron DSA*dn*, con un 40 % menos en la tasa de supervivencia del injerto a los diez años, comparada con la de pacientes sin DSA*dn*^13^.

Con base en las evidencias existentes hasta el momento se puede sugerir que, independientemente de la función inicial del injerto, la detección de DSA constituye un marcador importante para el seguimiento del estado inmunológico del injerto renal ^9^^,^^10^.

En el cuadro 1 se presentan los criterios actualmente utilizados en nuestro país y validados internacionalmente para el diagnóstico del rechazo mediado por anticuerpos (*antibody-mediated rejection*) (3,4,8). Por otra parte, los hallazgos histopatológicos para el diagnóstico del rechazo agudo y crónico, así como del rechazo celular o mediado por anticuerpos, se estratifican según los criterios de Banff, clasificación mundialmente aceptada pero no exenta de dificultades, pues no es 100 % reproducible entre diferentes observadores ^3^.

**Cuadro 1:** Criterios diagnósticos de rechazo mediado por anticuerpos (deben presentarse tres por lo menos)

• Evidencia clínica de la disfunción aguda del injerto
• Evidencia histológica del daño agudo del injerto: inflamación microvascular, capilaritis, arteritis de la íntima o transmural, y microangiopatía trombótica y necrosis tubular aguda en ausencia de otras causas
• Evidencia inmunopatológica por acción de anticuerpos: depósito difuso o focal de C4d en capilares peritubulares o de anticuerpos o C3 en las arterias, o activación endotelial (aumento de la expresión de mRNA de genes endoteliales (ENDAT) o presencia de células adhesivas y proliferativas en el endotelio capilar o glomerular (células CD31+ ki67+)
• Evidencia serológica de presencia de anticuerpos anti-HLA u otros anticuerpos específicos de donante en el momento de la biopsia

En el estudio histopatológico de las biopsias renales, es difícil reconocer el rechazo mediado por anticuerpos y se registra variabilidad entre observadores en los hallazgos morfológicos propuestos, además de que los anticuerpos no suelen detectarse por inmunofluorescencia indirecta (IFI). Teniendo en cuenta que el principal blanco del rechazo mediado por anticuerpos es el endotelio, el patólogo se enfoca en buscar lesiones en la microvasos (capilares), cambios en la morfología o causados por inflamación del capilar glomerular (glomerulitis-g), marginación leucocitaria (principalmente neutrófilos) con capilaritis peritubular, microangiopatía trombótica y cambios inducidos por isquemia isquemia, similares a los encontrados en la necrosis tubular aguda ^3^^,^^7^^,^^14^.

A partir de los años 90, se implementó la búsqueda de depósitos de la fracción C4d en capilares peritubulares mediante IFI. El C4d es un marcador específico (93-96 %) pero con sensibilidad muy variable (20-88 %) para demostrar la presencia de anticuerpos fijadores de complemento en el endotelio. Este aspecto se tuvo en cuenta en el consenso de Banff del año 2011, luego de la publicación del estudio BIFQUIT, sobre el control de calidad en la detección de C4d en las biopsias de los injertos renales ^10^.

El tema se ha discutido extensamente en la literatura especializada ^3^^,^^7^^,^^14^, y se ha observado que:

1. en los pacientes sensibilizados hay inflamación subclínica de la microcirculación (capilaritis o glomerulitis) que llevan al remodelado microvascular crónico o, lo que es lo mismo, a la glomerulopatía del trasplante y al aumento de la membrana basal de los capilares peritubulares;
2. el C4d es negativo en cerca del 20 % de los casos agudos de rechazo mediado por anticuerpos;
3. el C4d negativo en el rechazo mediado por anticuerpos es dos veces más frecuente, con un curso más lento e indolente del daño renal que cuando este es positivo, y
4. el rechazo crónico mediado por anticuerpos es independiente de la evidencia de C4d y responde primordialmente a la presencia de los DSA.

El consenso de Banff del 2013 incluyó en la nueva clasificación del rechazo la ausencia de C4d, dejando claro que, para el diagnóstico del rechazo mediado por anticuerpos, se requiere evidencia histológica de la interacción de los anticuerpos con el endotelio (no necesariamente positivo para C4d) y evidencia serológica de la presencia de aloanticuerpos circulantes ^3^^,^^14^, los cuales pueden ser anticuerpos anti-HLA o anti-endoteliales, y cuya detección en suero se recomienda el consenso de Banff del 2017.

Sin embargo, a pesar de que existe un consenso en los criterios clínicos y en las pruebas para la detección y el diagnóstico del rechazo agudo celular, el diagnóstico del rechazo mediado por anticuerpos y la detección del inicio del rechazo crónico siguen siendo un reto para los diferentes grupos de trasplante ^15^.

La implementación de un protocolo de seguimiento del paciente con trasplante renal basado en criterios inmunológicos podría constituir una herramienta para la detección temprana del rechazo mediado por anticuerpos y, en consecuencia, para la implementación de estrategias de manejo que prevengan o demoren al máximo la aparición del rechazo crónico o la pérdida del órgano trasplantado ^13^^,^^16^^,^^17^.

## Seguimiento inmunológico después del trasplante

La determinación de los DSA en el momento del trasplante permite clasificar a los pacientes según su riesgo de rechazo mediado por anticuerpos ^15^^,^^18^^-^^20^, así, los pacientes con riesgo alto son aquellos positivos para anticuerpos anti- HLA específicos de donante (DSA) en el momento del trasplante y con prueba cruzada (XM) de citotoxicidad negativa para linfocitos T en suero tomado en los tres meses previos (positivos para DSA y negativos con la XM del CDC); los pacientes con riesgo intermedio son aquellos con anticuerpos anti-HLA positivos pero sin DSA y con prueba cruzada de citotoxicidad positiva para linfocitos T en sueros tomados más de tres meses antes de la prueba, pero negativa en suero tomado en los tres meses previos (positivos para anticuerpos anti-HLA, pero negativos para DSA, y positivos con la XM-CDC en suero tomado antes de los tres meses previos, pero negativos en suero tomado en los tres meses previos), en tanto pacientes con riesgo bajo son aquellos con anticuerpos anti-HLA negativos y prueba cruzada de citotoxicidad negativa para linfocitos T en sueros tomados más de tres meses antes de la prueba y en los tomados en los tres meses previos (negativos para anticuerpos anti-HLA y negativos en la XM-CDC en sueros tomados más de tres meses antes de la prueba y en los tomados en los tres meses previos). Estos parámetros se resumen en el cuadro 2.

**Cuadro 2:** Parámetros para determinar el riesgo inmunológico de rechazo mediado por anticuerpos según los resultados de las pruebas de histocompatibilidad

Riesgo inmunológico de rechazo mediado por anticuerpos	Resultados en las pruebas de histocompatibilidad			
	Anticuerpos anti-HLA específicos de donante (DSA)	Anticuerpos anti-HLA no DSA	Prueba cruzada de citotoxicidad para linfocitos T en suero actual*	Prueba cruzada de citotxicidad para linfocitos T en suero histórico^†^
Alto	Positivo	Positivo o negativo	Negativo	Negativo
Intermedio	Negativo	Positivo	Negativo	Positivo
Bajo	Negativo	Negativo	Negativo	Negativo

^*^ Suero actual se refiere al tomado en los últimos tres meses.

^†^ Suero histórico se refiere al tomado más de tres meses antes de la prueba.

Durante el seguimiento inmunológico del trasplante renal, se pueden emplear pruebas para la detección temprana del rechazo celular, entre ellas, las de medición del aumento de la proteína CD30 soluble (sCD30) y la secreción de IFN-y por los linfocitos T (EliSpot), pero estas no se usan de forma generalizada y la comunidad internacional no las acepta suficientemente para este fin. Sin embargo, para la detección precoz de los cambios del injerto inducidos por anticuerpos, se emplea el seguimiento mediante medición de los DSA en el suero del paciente, un recurso que ha sido ampliamente difundido y validado por la mayoría de los laboratorios de histocompatibilidad en el mundo ^14^^,^^21^^,^^22^.

Las pruebas actualmente avaladas por la *Food and Drug Administration* (FDA) de los Estados Unidos y el Instituto Nacional de Vigilancia de Medicamentos y Alimentos (Invima) en Colombia para la detección de los DSA, son la cuantitativa de detección de anticuerpos anti-HLA de clases I y II (panel reactivo de anticuerpos, PRA) y la de anticuerpos anti-HLA de antígeno aislado de clases I y II. Esta última, de gran sensibilidad y especificidad para caracterizar los anticuerpos anti-HLA, incluso en pacientes muy sensibilizados, depende de la disponibilidad de información antes del trasplante (HLA del donante). Con ella se puede determinar si los anticuerpos son específicos de donante o no (prueba cruzada virtual), y si son *de novo* o estaban presentes antes del trasplante ^23^^-^^26^. Otras pruebas para el diagnóstico *in vitro* que aún no están disponibles en el país, incluyen la técnica cualitativa para la detección de anti-HLA mediante la tecnología xMAP^™^ de Luminex (prueba cruzada) y la medición de la expresión de los mRNA de genes endoteliales (*endothelial-associated transcripts,* ENDAT) ^27^^,^^28^.

La detección de DSA en el seguimiento de los pacientes con trasplante también ha permitido determinar aspectos clave para su evaluación histológica e inmunológica ^17^. El papel de las biopsias hechas por protocolo ha sido cuestionado por algunos grupos de trasplante, pero hay evidencia de su utilidad por indicación en pacientes con presencia de DSA antes del trasplante (alto riesgo), en aquellos con DSA*dn* después del trasplante, tras cambios significativos en el tratamiento inmunosupresor y frente a la sospecha de que el paciente no está cumpliendo con el tratamiento ^3^^,^^13^. En el algoritmo que proponemos para el seguimiento inmunológico después del trasplante, se tuvo en cuenta esta evidencia al incluir el estudio anatomopatológico del injerto renal para el diagnóstico y seguimiento del rechazo después del tratamiento ^15^.

El papel de la detección de DSA después del trasplante también ha sido cuestionado por algunos debido a que, a simple vista, aumenta los costos del seguimiento de los pacientes con trasplante y todavía no se sabe con certeza el verdadero impacto de dar tratamiento a los pacientes cuando solo están presentes dichos anticuerpos. Sin embargo, son suficientes las evidencias científicas aquí expuestas que indican que los DSA aparecen en todos los injertos, temprana o tardíamente, y que, en todos los casos, su detección tiene implicaciones para la supervivencia del injerto a largo plazo, uno de los retos actuales de los grupos de trasplante; además, son una garantía de calidad en la práctica clínica y de mejoramiento de la calidad de vida y la productividad de las personas, lo que justifica la inversión económica del sistema de salud ^3^^,^^5^^,^^15^^,^^17^^,^^23^^-^^26^.

Además del seguimiento clínico para conocer el éxito del tratamiento del rechazo agudo, debe solicitarse la biopsia (con C4d para el rechazo mediado por anticuerpos) y vigilar los DSA ^3^^,^^13^^,^^15^. Se considera que es ideal tomar una nueva biopsia a los tres a cinco días de finalizado el tratamiento, y una nueva muestra de suero a los siete a diez días. Los resultados esperados son la disminución de los cambios histológicos iniciales asociados con el rechazo agudo, la desaparición de los DSA presentes antes del tratamiento o la disminución de la intensidad media de su fluorescencia (MFI) en más de un 25 % ^3^^,^^15^.

Con los elementos planteados, se puede concluir que el seguimiento de los DSA es indispensable para el diagnóstico precoz del rechazo mediado por anticuerpos y la intervención farmacológica, en aras de evitar la aparición del rechazo crónico que, como ya se sabe, lleva a la pérdida de funcionalidad del órgano trasplantado.

**Figura 1:**
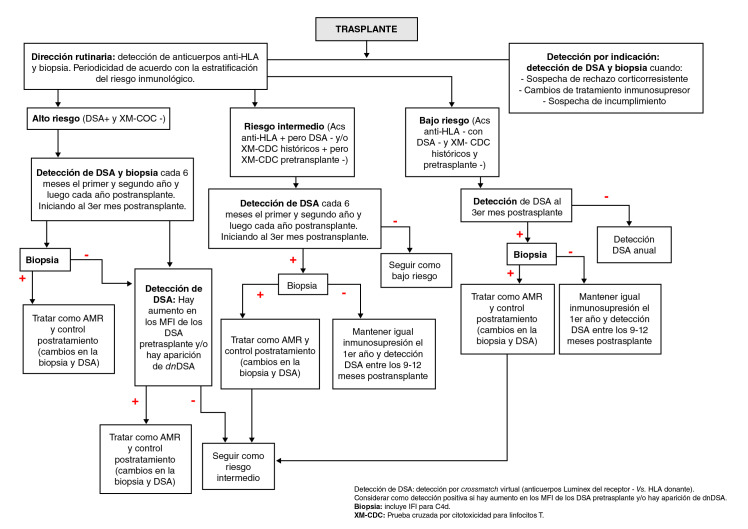
Algoritmo propuesto para el seguimiento después de un trasplante renal. En la interpretación de los resultados de las pruebas de histocompatibilidad, deben considerarse algunos factores de la condición clínica de cada paciente que pueden interferir en la interpretación de los resultados de anticuerpos, por ejemplo, presencia de HIV, del virus de la hepatitis C, de enfermedades autoinmunitarias, de diabetes, uso de medicamentos anti-CD20 (rituximab) o inmunoglobulina G endovenosa (IVIG) o plasmaféresis como tratamiento contra el rechazo.

En el algoritmo que se sugiere (figura 1), se establece cómo deber ser el seguimiento inmunológico del paciente con trasplante renal y las correspondientes conductas según los resultados obtenidos. Es importante resaltar que, ocasionalmente, hay pacientes con cambios histológicos o clínicos pero sin DSA, lo que obliga a pensar en la presencia de otros aloanticuerpos diferentes a los anticuerpos anti-HLA (anti-receptor de angiotensina II de tipo 1, AT1R, los anti-vimentina, los anti-MICA, etc.) y a los anticuerpos anti-HLA no fijadores de complemento o de baja reacción, o a considerar la posibilidad de que el injerto absorba los anticuerpos y no puedan detectarse en sangre periférica ^15^^,^^29^^,^^30^. La ausencia de DSA detectables en sangre periférica en el momento de una biopsia con hallazgos positivos para rechazo mediado por anticuerpos, no descarta el diagnóstico y la necesidad del tratamiento.
